# Controlling the Isothermal Crystallization of Isodimorphic PBS-*ran*-PCL Random Copolymers by Varying Composition and Supercooling

**DOI:** 10.3390/polym12010017

**Published:** 2019-12-20

**Authors:** Maryam Safari, Agurtzane Mugica, Manuela Zubitur, Antxon Martínez de Ilarduya, Sebastián Muñoz-Guerra, Alejandro J. Müller

**Affiliations:** 1 POLYMAT and Polymer Science and Technology Department, Faculty of Chemistry, University of the Basque Country UPV/EHU, Paseo Manuel de Lardizabal 3, 20018 Donostia-San Sebastián, Spain; maryam.safari@polymat.eu (M.S.); agurtzane.mugica@ehu.es (A.M.); 2 Chemical and Environmental Engineering Department, Polytechnic School, University of the Basque Country UPV/EHU, Plaza Europa 1, 20018 Donostia-San Sebastián, Spain; manuela.zubitur@ehu.eus; 3 Departament d’Enginyeria Química, Universitat Politècnica de Catalunya, ETSEIB, Diagonal 647, 08028 Barcelona, Spain; antxon.martinez.de.ilarduia@upc.edu (A.M.d.I.); sebastian.munoz@upc.edu (S.M.-G.); 4 IKERBASQUE, Basque Foundation for Science, María Díaz Haroko Kalea, 3, 48013 Bilbao, Spain

**Keywords:** isodimorphism, random copolymers, crystallization, nucleation, growth rate

## Abstract

In this work, we study for the first time, the isothermal crystallization behavior of isodimorphic random poly(butylene succinate)-*ran*-poly(ε-caprolactone) copolyesters, PBS-*ran*-PCL, previously synthesized by us. We perform nucleation and spherulitic growth kinetics by polarized light optical microscopy (PLOM) and overall isothermal crystallization kinetics by differential scanning calorimetry (DSC). Selected samples were also studied by real-time wide angle X-ray diffraction (WAXS). Under isothermal conditions, only the PBS-rich phase or the PCL-rich phase could crystallize as long as the composition was away from the pseudo-eutectic point. In comparison with the parent homopolymers, as comonomer content increased, both PBS-rich and PCL-rich phases nucleated much faster, but their spherulitic growth rates were much slower. Therefore, the overall crystallization kinetics was a strong function of composition and supercooling. The only copolymer with the eutectic composition exhibited a remarkable behavior. By tuning the crystallization temperature, this copolyester could form either a single crystalline phase or both phases, with remarkably different thermal properties.

## 1. Introduction

Biocompatible and biodegradable polymers are being developed for a wide range of applications due to their potential to solve the environmental concerns caused by traditional nondegradable plastics [1,2,3,4]. Among the biodegradable polymers that have been most intensively studied are aliphatic polyesters such as Poly(glycolide) (PGA), Poly(L-lactide) (PLLA), Poly(ethylene succinate) (PES), Poly(butylene succinate) (PBS), and Poly(ε-caprolactone) (PCL) [5,6]. Although aliphatic polyesters have been used for many years in industrial, biomedical, agricultural, and pharmaceutical applications, there is still room for many improvements [7,8]. The synthesis of random copolyesters, using biobased comonomers, can overcome some of the drawbacks of biodegradable polyesters, such as slow biodegradation rate (due to high crystallinity degrees) and undesirable mechanical properties [9,10,11].

The properties of crystallizable random copolymers constituted by two semicrystalline parent components have been recently reviewed [12]. Depending on their ability to share crystal lattices, three different cases have been reported [12,13,14]: (a) total comonomer exclusion occurs when the chemical repeat units are very different and the crystal lattice of each one of the components cannot tolerate the presence of the other; (b) total comonomer inclusion or isomorphic behavior can only be obtained in cases where the components can cocrystallize in the entire composition range (as their chemical structures are very similar), forming a single crystal structure [15,16]; (c) an intermediate and complex case, where a balance between comonomer inclusion and exclusion occurs, leading to isodimorphic copolymers. 

In isodimorphic random copolymers, at least one of the two crystalline phases includes some repeat units of the minor component in its crystal lattice. When the melting point is plotted as a function of composition a pseudo-eutectic behavior is commonly observed, where, on each side of the pseudo-eutectic point, only the crystalline phase of the major component is formed, which may contain a limited amount of the minor comonomer chains included in the crystal lattice [12].

In our previous works [10,17], we have synthesized and studied the morphology and crystallinity of poly (butylene succinate-*ran*-caprolactone) (PBS-*ran*-PCL) copolyesters. In situ wide angle X-ray scattering (WAXS) indicated that changes were produced in the crystalline unit cell dimensions of the dominant crystalline phase. In addition, differential scanning calorimetry (DSC) measurements showed that all copolymers could crystallize, regardless of composition, and their thermal transitions temperatures (i.e., *T_c_* and *T_m_*) went through a pseudo-eutectic point when plotted as a function of composition. Therefore, all this evidence demonstrated an isodimorphic behavior. At the pseudo-eutectic composition, both PBS-rich and PCL-rich phases can crystallize [10,17]. 

In the current work, we perform a detailed isothermal crystallization study of PBS-*ran*-PCL copolymers to determine the nucleation and crystallization kinetics of the copolyesters and study the influence of composition on the crystallization kinetics. This information is very important as it allows tailoring the properties of random copolymers as well as their applications. The analysis of the isothermal crystallization kinetics of PBS-*ran*-PCL was performed using differential scanning calorimetry (DSC), polarized light optical microscopy (PLOM), and in situ wide angle X-ray scattering (WAXS). 

## 2. Materials and Methods

### 2.1. Synthesis

The PBS-*ran*-PCL copolymers were synthesized by a two-stage melt-polycondensation reaction. First, transesterification/ROP reaction of dimethyl succinate (DMS), 1,4-butanediol (BD), and ε-caprolactone (CL), and then polycondensation at reduced pressure, as reported in detail previously [17]. Samples are denoted in an abbreviated form, e.g., BS_x_CL_y_, indicating the molar ratio of each component determined by ^1^H-NMR, as subscripts (x and y). Table 1 shows molar composition, number- and weight-average molar mass and thermal transitions of the isodimorphic random copolyesters under study in this work.

### 2.2. Polarized Light Optical Microscopy (PLOM)

A polarized light optical microscope, Olympus BX51 (Olympus, Tokyo, Japan), equipped with an Olympus SC50 digital camera and with a Linkam-15 TP-91 hot stage (Linkam, Tadworth, UK) (coupled to a liquid nitrogen cooling system) was used to observe spherulites nucleation and growth. Films with around 100 μm thickness were prepared by melting the samples in between two glass slides. For the isothermal experiments, the conditions were very similar to those employed during DSC measurements. The samples were heated to 30 °C above their melting point to erase their thermal history, and then were rapidly cooled from the melt at 60 °C/min to the selected isothermal crystallization temperature, *T_c_*. Then, the sample was kept at *T_c_* while the spherulites appeared and grew. To better compare the compositions in a fixed common crystallization temperature, *T_c_* values were chosen at the same supercooling degree for PBS-rich compositions at Δ*T* = 40, 38, 36, 34, and 32 °C and for PCL-rich compositions at Δ*T* = 40, 39, 38, 37, and 36 °C. The supercooling was calculated from the equilibrium melting temperatures determined by Hoffman–Weeks extrapolations after isothermal crystallization in the DSC, see below.

### 2.3. Differential Scanning Calorimetry (DSC)

Isothermal differential scanning calorimetry experiments were performed using a Perkin Elmer 8500 calorimeter equipped with a refrigerated cooling system Intracooler 2P, under a nitrogen atmosphere (with a flow of 20 mL/min) and calibrated with high purity indium and tin standards. The weight of the samples was about 5 mg and samples were hermetically sealed in standard aluminum pans. 

To investigate the overall crystallization kinetics, an isothermal protocol was applied. First, the minimum isothermal crystallization temperature *T_c,min_* was determined by trial and error following Müller et al. [18,19]. Samples were quenched to *T_c_* values (estimated from the nonisothermal DSC runs) at 60 °C/min and then immediately reheated at 20 °C/min up to temperatures above the melting point of the crystalline phase involved. If any latent melting enthalpy is detected, this means that the sample was able to crystallize during the cooling to *T_c_*, therefore, this *T_c_* value cannot be used as *T_c,min_* and a higher *T_c_* value is explored. 

After the *T_c_* range was determined, the isothermal crystallization experiments were performed, closely following the procedure suggested by Lorenzo et al. [19]: (I) heating from room temperature to 30 °C above their melting point at 10 °C/min; (II) holding the sample for 3 min at that temperature to erase thermal history; (III) quenching the sample to a predetermined crystallization temperature (*T_c_*) at 60 °C/min. *T_c_* was in the range between −3 and 90 °C depending on composition; (IV) isothermal crystallization until maximum saturation; (V) heating from *T_c_* to 30 °C above the melting point of the sample at 10 °C/min, to record the melting behavior after the isothermal crystallization. This final melting run provided the values of apparent melting points that were employed to perform the Hoffman–Weeks extrapolation to calculate the equilibrium melting temperature of each material.

### 2.4. Simultaneous WAXS Synchrotron Measurements

To study the crystal structure during isothermal crystallization at the pseudo-eutectic point, the BS_45_CL_55_ sample was examined by in situ WAXS performed at beamline BL11-NCD at the ALBA Synchrotron radiation facility, Cerdanyola del Vallés, Barcelona, Spain. The samples in DSC aluminum pans were placed in a Linkam THMS-600 stage coupled to a liquid nitrogen cooling system. WAXS scans were taken periodically every 30 s during the isothermal crystallization. The energy of the X-ray source was 12.4 keV (*λ* = 1.0 Å). In the WAXS configuration, the sample-detector, Rayonix LX255-HS with an active area of 230.4 × 76.8 mm (pixel size: 44 μm^2^) distance employed was 15.5 mm with a tilt angle of 27.3°. The scattering vector was calibrated using chromium (III) oxide for WAXS experiments.

## 3. Results

We have studied previously [17] the nonisothermal crystallization behavior of the same PBS-ran-PCL random copolymers employed in this work. The results demonstrated that these copolymers exhibit an isodimorphic behavior.

**Figure 1:**
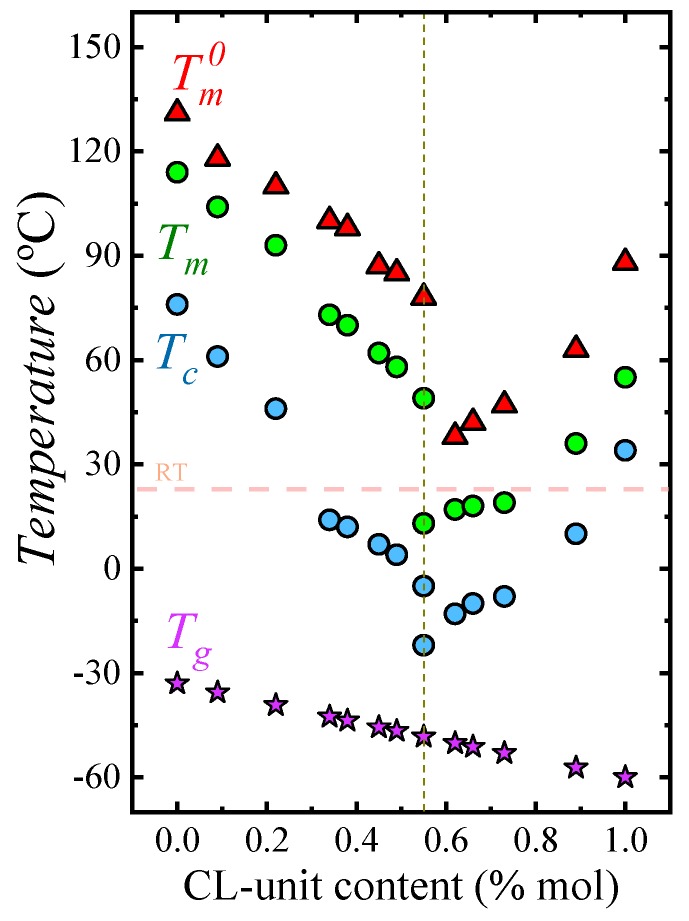
Phase diagram based on data published in [17] on the nonisothermal crystallization of the PBS-*ran*-PCL copolymers under study. Additionally, equilibrium melting temperatures obtained in the present work by Hoffman–Weeks analysis of isothermally obtained data, are also included. The dashed vertical line indicates the pseudo-eutectic point. The dashed horizontal line indicates an arbitrary room temperature value.

Figure 1 presents a phase diagram for the PBS-ran-PCL system. These random copolymers exhibit a single-phase melt and a single glass transition temperature, as expected for random copolymers. Upon cooling from the melt, the materials are capable of crystallizing in the entire composition range, in spite of being random, as demonstrated by NMR studies [17]. The copolymers display a pseudo-eutectic point at the composition BS_45_CL_55_. This BS_45_CL_55_ copolymer is the only one in the series that can form two crystalline phases upon cooling from the melt, i.e., a PBS-rich phase and a PCL-rich phase (as evidenced earlier by WAXS and DSC [17]), hence the two melting point values reported in Figure 1 for this composition. To each side of the pseudo-eutectic point, a single crystalline phase is formed, either a PBS-rich phase (i.e., left-hand side of the eutectic) or a PCL-rich phase (i.e., right-hand side of the eutectic), with crystalline unit cells resembling those of PBS and PCL respectively.

In the present work, we performed isothermal crystallization studies and calculated the equilibrium melting temperatures ($T_{m}^{0}$) of homopolymers and copolymers by employing the Hoffman–Weeks extrapolation. Examples of Hoffman–Weeks plots can be found in Figure SI-3, while Figure 1 reports the variation of the equilibrium melting temperatures obtained with composition. The $T_{m}^{0}$ values show a similar trend with composition as the apparent melting peak temperatures determined by DSC during nonisothermal experiments, and they also display a pseudo-eutectic point. These $T_{m}^{0}$ values will be employed throughout this paper, as they are needed to fit the Lauritzen and Hoffman nucleation and crystallization theory to analyze the experimental data.

The phase diagram shown in Figure 1 illustrates the versatility of isodimorphic copolymers. It is well known that the optimal mechanical properties in terms of ductility and toughness of thermoplastic semicrystalline materials are generally observed at temperatures in between *T_g_* and *T_m_*. Thanks to random copolymerization, the copolymers exhibit a single *T_g_* value that is independent of the melting point of the phase (or phases in the case of the composition at the pseudo-eutectic point) that is able to crystallize. This remarkable behavior provides a separate control of *T_g_* and *T_m_* which cannot be obtained in homopolymers. Additionally, as Figure 1 shows, depending on composition, the samples can be molten at room temperature or they can be semicrystalline. Such wide range of thermal properties can lead to fine tuning mechanical properties and crystallinities to tailor applications.

### 3.1. Nucleation Kinetics Studied by PLOM

Counting the number of spherulites in PLOM experiments is the usual way of obtaining nucleation data by assuming that each spherulite grows from one heterogeneous nucleus. In this work, we studied the nucleation kinetics by determining the nucleation density as a function of time by PLOM, from which nucleation rates can be calculated.

**Figure 2:**
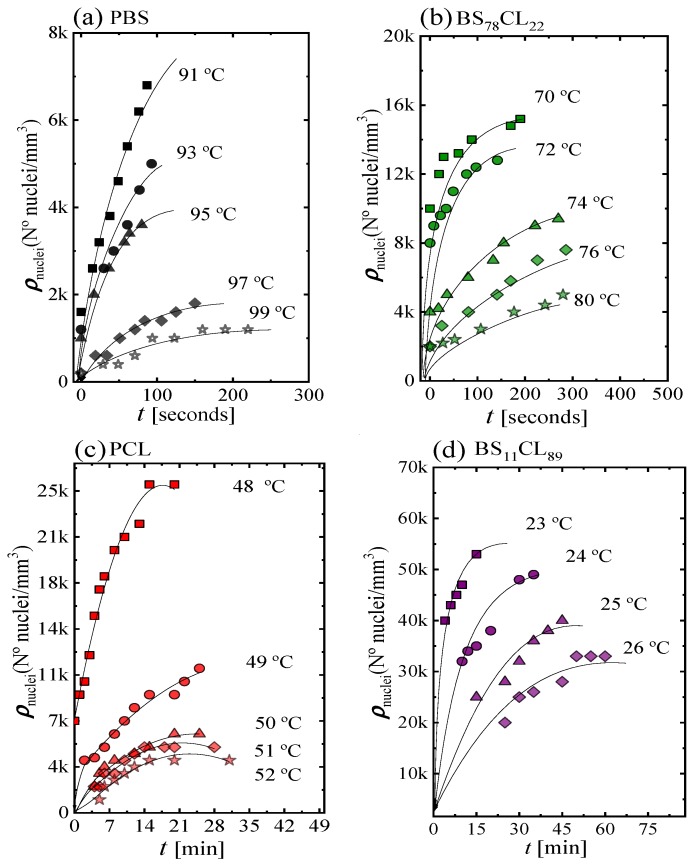
Nucleation kinetics data obtained by polarized light optical microscopy (PLOM): (**a**) PBS; (**b**) BS_78_CL_22_; (**c**) PCL; (**d**) BS_11_CL_89_. Nuclei density as a function of time at different crystallization temperatures for the indicated samples.

Figure 2 shows four examples of plots of the nucleation density ρ_nuclei_ (nuclei/mm^3^) as a function of time for neat PBS, neat PCL, and two sample copolymers. The rest of the data can be found in the Supplementary Information (Figure SI-1). The nucleation density increases almost linearly with time at short times, then it tends to saturate. The number of heterogeneous nuclei that are activated at longer times increases as nucleation temperature decreases, a typical behavior of polymer nucleation [20]. As expected, the nucleation density at any given time increases as *T_c_* decreases, because the thermodynamic driving force for primary nucleation increases with supercooling [21].

**Figure 3:**
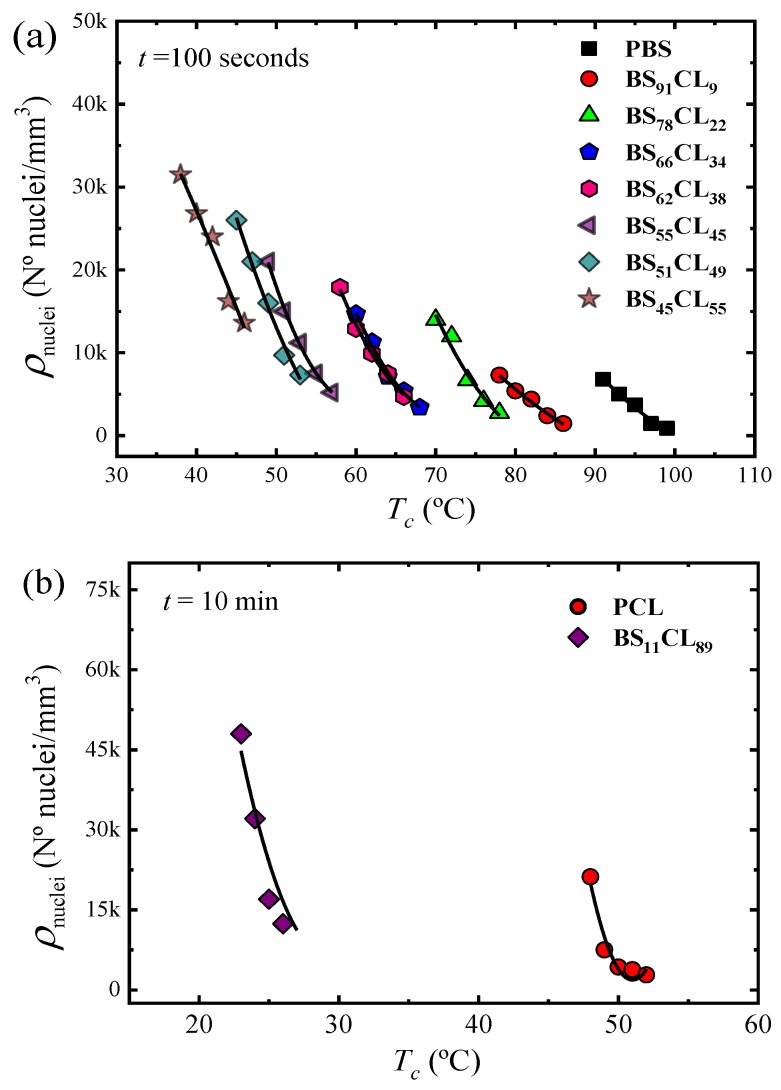
(**a**) Nuclei density during isothermal crystallization as a function of *T_c_* at a constant time of 100 s for PBS-rich compositions and (**b**) at 10 min for PCL-rich compositions.

Figure 3 shows plots of nucleation density versus temperature taken at a constant nucleation time of 100 s for neat PBS and PBS-rich copolymers (Figure 3a) and 10 min in the case of PCL and BS_11_CL_89_ copolymer (Figure 3b). 

PBS exhibits the lowest nucleation density of all samples, therefore, the largest spherulites (see Figure 5 below). As the amount of CL comonomer increases in the PBS-rich copolymers (Figure 3a), the nucleation density increases, as well as the supercooling needed for nucleation. In the case of PBS-rich copolymers, Figure 3a (and Figure SI-1 in the SI) shows nucleation data for seven different copolymers, with compositions ranging from 91% to 45% PBS.

The dependence of nucleation density on supercooling, can be observed in Figure SI-2. The data presented in Figure 3a can be reduced to a supercooling range between 32 and 40 °C, i.e., only 8 °C. This means that a large part of the horizontal shift in the curves of Figure 3a (spanning nearly 60 °C in crystallization temperature) is due to changes in supercooling. These changes are caused by the variations in equilibrium melting temperatures with composition (see Figure 1).

PCL has a higher nucleation density than PBS when compared at equal supercoolings (see Figure SI-2b). When a small amount of BS comonomer is incorporated, as in random copolymer BS_11_CL_89_, the nucleation density increases significantly (Figure 3b). Due to the very high nucleation density of the other PCL-rich composition copolymers (with higher amounts of PBS), it was impossible to determine their nucleation kinetics. Examples of the microspherulitic morphologies obtained for such PCL-rich copolymers can be observed in Figure 5 below.

It is interesting to note than in both sides of the pseudo-eutectic point (i.e., the PBS-rich side represented in Figure 3a and the PCL-rich side represented in Figure 3b, see also Figure 1), the copolymers exhibit higher nucleation density than their corresponding homopolymers. This behavior could be somewhat analogous to what has been observed in long-chain branched polylactides (PLLAs) [22] or long-chain branched polypropylenes (PPs) with respect to linear analogs [23]. The interruption of crystallizable linear sequences with defects has been reported to increase nucleation density although the reasons are not clear. In the present case, the linear crystallizable sequence of PBS, for instance, is being changed by the introduction of randomly placed PCL repeat units. Even though the random copolyesters can form a single phase in the melt, there may be at the segmental level, some preference for PBS-PBS local chain segmental contacts in comparison to less favorable PBS-PCL contacts. We speculate that this may drive the enhancement of nucleation, but more in-depth studies would be needed to ascertain the exact reason for this behavior.

The Fisher–Turnbull nucleation theory [24] can be used to quantify the activation free energy of primary nucleation. This theory gives the steady-state rate of primary nucleation per unit volume and time, *I* = dN/dt, for a heterogeneous nucleation process on a preexisting flat surface (or heterogeneous nucleus) as:

(1) $$logI=logI_{0}-\frac{\Delta F^{*}}{2.3kT}-\frac{16\sigma \sigma_{e}\left(\Delta \sigma \right)T_{m}^{{}^{\circ}2}}{2.3kT{\left(\Delta T\right)}^{2}{\left(\Delta H_{v}\right)}^{2}},$$

where *I*_0_ is related to the diffusion of polymeric segments from the melt to the nucleation site, ΔF* is a parameter proportional to the primary nucleation free energy, and *σ* and *σ_e_* are the lateral and fold surface free energies, respectively. Δ*T* is the supercooling defined as Δ*T* = $T_{m}^{0}$ − $T_{c}$ and $T_{m}^{0}$ is the equilibrium melting point. Δ*σ* is the interfacial free energy difference, given by:

∆*σ* = *σ* + *σ_s/c_* − *σ_s/m_*, (2)

in which *σ_s/c_* is the crystal-substrate interfacial energy and *σ_s/m_* is the melt-substrate interfacial energy. Therefore, Δ*σ* can be considered proportional to the surface tension properties of the substrate, polymer crystal and polymer melt. The interfacial free energy difference is a convenient way to express the nucleating ability of the substrate towards the polymer melt.

In this work, the values of $T_{m}^{0}$ (listed in Table SI-1 and plotted in Figure 1) were obtained by isothermal crystallization DSC experiments followed by Hoffman–Weeks extrapolations (see Figure SI-3). Δ*H_v_* is the volumetric melting enthalpy (J/m^3^) and it was estimated by $\Delta H_{V}=\Delta H_{m}^{0\;}\times \rho$, so that *ρ* = 1.26 g/cm^3^ and $\Delta H_{m}^{0}$ = 213 J/g for neat PBS [25] and *ρ* = 1.14 g/cm^3^ and $\Delta H_{m}^{0}$ = 139.5 J/g for neat PCL [26]. 

In this work, we employed the value of $\Delta H_{m}^{0}$ = 213 J/g for neat PBS and PBS-rich phase composition that has been recently obtained by some of us [25]. This value was determined employing a combined DSC and X-ray diffraction method using isothermal crystallization data. This experimentally extrapolated value is higher than that of $\Delta H_{m}^{0\;}$ = 110 J/g, estimated empirically by the group contribution method [27], but very close to the value of 210 J/g reported by Papageorgiou et al. [28].

**Figure 4:**
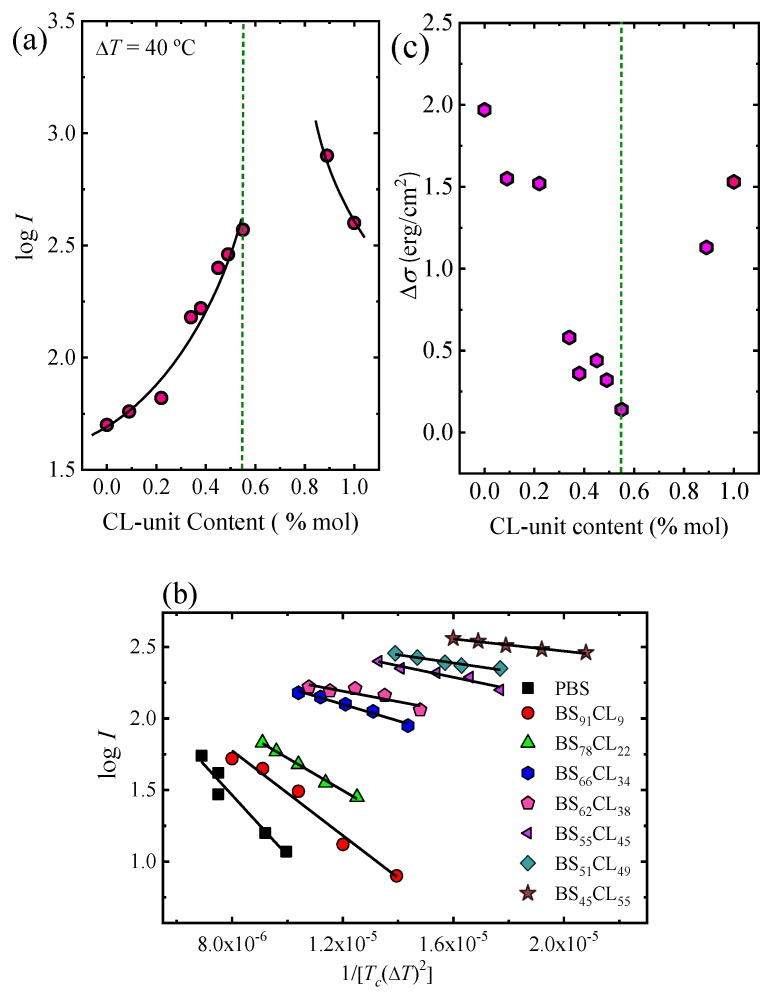
Nucleation rate (*I*) data: (**a**) log I as a function of copolymer composition, expressed as mol % of ε-caprolactone (CL) units, taken at a constant supercooling of Δ*T* = 40 °C. The segmented vertical line is drawn to indicate the pseudo-eutectic composition. (**b**) Plot of *log I* versus 1/*T_c_*(Δ*T*)^2^ for PBS-rich compositions. The black lines represent fittings to the Turnbull–Fisher equation (Equation (1)). (**c**) Interfacial free energy difference (Δ*σ*) as a function of composition. The segmented vertical line is drawn to indicate the pseudo-eutectic composition.

The values of the nucleation rate I were calculated from the initial slope (i.e., at short measurement times, where linear trends were obtained) of the plots shown in Figure 2 and Figure SI-1. Figure 4a shows log I as a function of CL-unit molar fraction for a constant supercooling of Δ*T* = 40 °C. The nucleation rate strongly depends on copolymer composition. Adding a comonomer randomly along the chain to either PBS or PCL largely increases the nucleation rate. In the PBS-rich composition side (to the left of the pseudo-eutectic point signaled by a vertical line in Figure 4a) the nucleation rate increases up to 7.5 times with respect to neat PBS, as the amount of PCL units in the random copolymer increases. Neat PCL nucleates faster than neat PBS. In the PCL-rich composition side, only one copolymer was measured (whose nucleation rate increased two-fold with respect to neat PCL), as increasing PBS content towards the pseudo-eutectic point increased nucleation rate so much that measurements were no longer possible. 

Figure 4b shows the Turnbull–Fisher plots for PBS and PBS-rich compositions based on Equation (1). Turnbull–Fisher plots for PCL and BS_11_CL_89_ copolymer are presented in the supplementary information (Figure SI-4). The nucleation data can be successfully fitted with the linearized version of Equation (1). From the slope, a value of the interfacial free energy difference (Δ*σ*) can be obtained.

Small values of Δ*σ* are indicative of good nucleation efficiency since a lower amount of interfacial energy is required to form the crystal–substrate interface. Table 2 reports a value of Δ*σ* for PBS equal to 1.97 erg/cm^2^. As seen in Figure 4c (and Table 2), this interfacial free energy difference progressively decreases in the copolymers as the amount of CL comonomer increases, indicating that the primary nucleation process is facilitated by copolymerization with PCL until the pseudo-eutectic point is reached. On the right-hand side of the pseudo-eutetic point in Figure 4c, PCL has a Δ*σ* value of 1.53 erg/cm^2^, which is, as expected, smaller than that of PBS, as PCL has a larger nucleation density at equivalent supercoolings than PBS. The copolymer B_11_CL_89_ shows an even smaller value of Δ*σ*, as the incorporation of PBS in the copolymer increases its nucleation capacity.

### 3.2. Kinetics of Superstructural Growth (Secondary Nucleation) by PLOM

**Figure 5:**
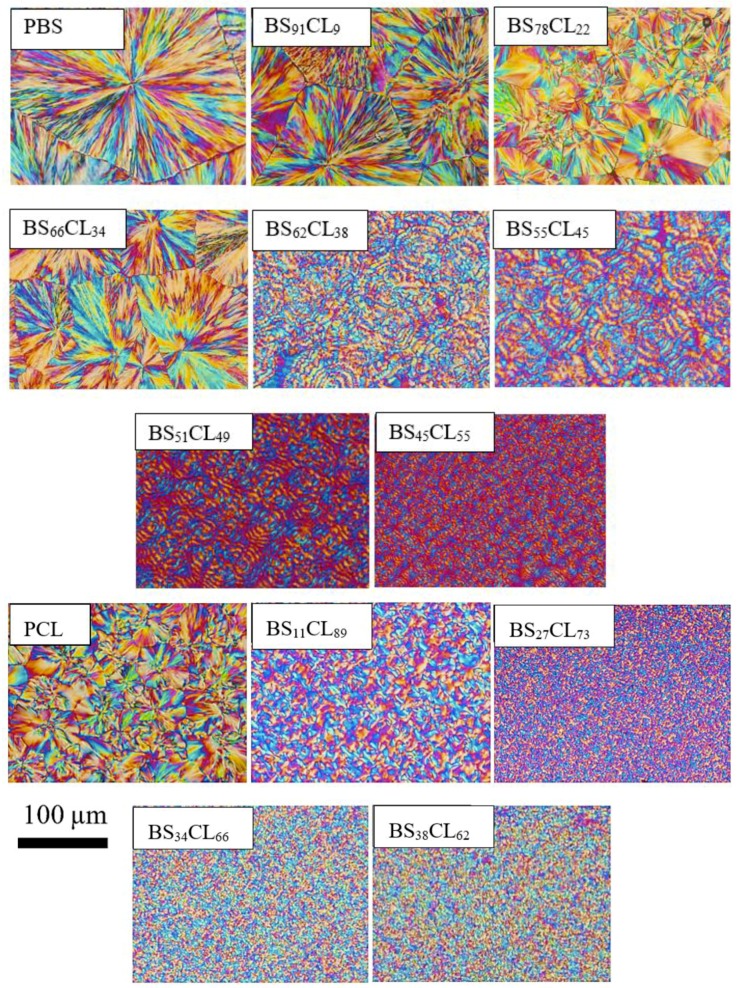
PLOM micrographs after isothermal crystallization at Δ*T* = 40 °C for the indicated samples.

PBS, PCL, and all the random copolymers prepared in this work exhibited spherulitic superstructural morphologies. Examples of the spherulites obtained at a constant supercooling value of 40 °C can be observed in Figure 5. Both PBS and PCL exhibited well-developed spherulites without banding. PBS-rich copolymers that contain more than 34% PCL exhibit clear banding. This is consistent with previous works indicating that the addition of diluents (for PBS-rich compositions, crystallization occurs while PCL chains are in the liquid state) to several polyesters induces banding [29,30].

Isothermal crystallization experiments were performed to follow the growth of spherulites as a function of time using PLOM. The growth rate was calculated from the slope of spherulite radius versus time plots, which were always observed to be highly linear [18,31]. 

**Figure 6:**
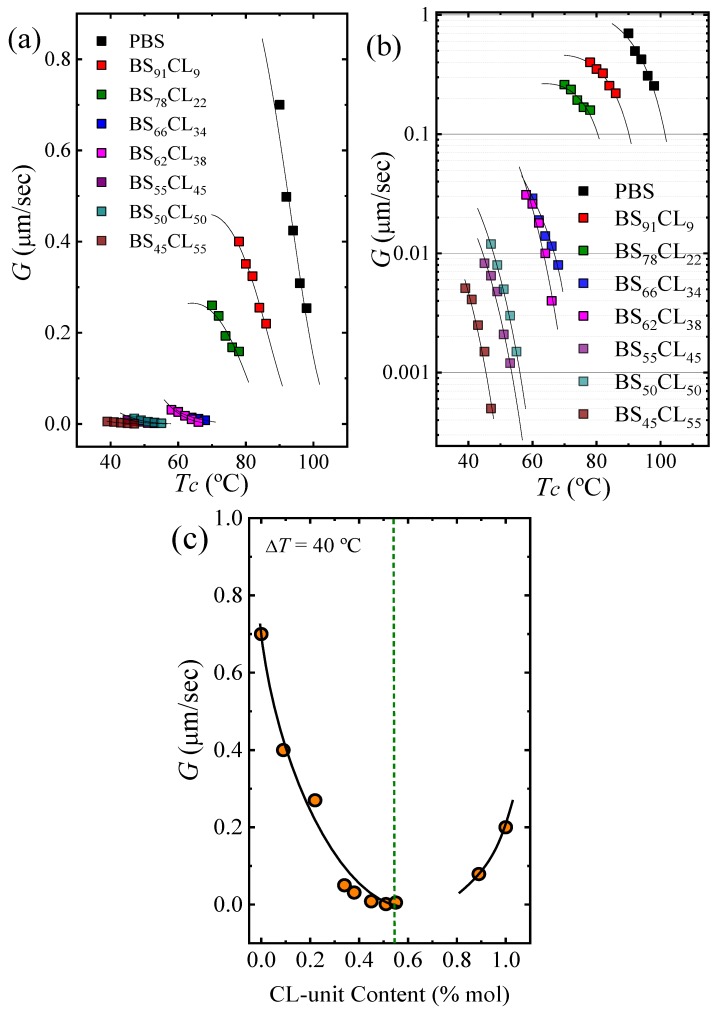
Spherulitic growth rates determined by PLOM. (**a**) Growth rate, G, as a function of *T_c_*. (**b**) Same data as in (**a**) but G is plotted on a logarithmic scale. The black solid lines are fits to the experimental data performed with the Lauritzen and Hoffman theory (L-H). (**c**) G versus CL-unit content at Δ*T* = 40 °C. The black line is an arbitrary polynomial fit drawn to guide the eye.

The experimental growth rates are plotted as a function of the isothermal crystallization temperatures employed in Figure 6a with a linear scale and in Figure 6b with a log scale, so that differences in G values for PBS-rich samples with PCL contents larger than 22% are observed. The incorporation of PCL repeat units in the random copolymers have a dramatic influence on the growth rate of the PBS-rich phase spherulites, as G decreases up to 3.5 orders of magnitude (Figure 6b). The decrease in G values with comonomer incorporation for the PBS rich copolymers is due to two reasons. Firstly, as in any isodimorphic copolymer, there is a competition between inclusion and exclusion of repeat units within the PBS crystal lattice, where exclusion typically predominates. Secondly, incorporation of PCL repeat units in the copolymer chains reduces *T_g_* values (as shown in Figure 1), thereby causing a plasticization effect on the PBS-rich phase. In the case of the PCL-rich compositions, the spherulitic growth rate was determined for only one copolymer (i.e., BS_11_CL_89_), as in the other cases, as pointed out above, the nucleation rate and nucleation density were so high, that it was impossible to measure the extremely fast growth of very small spherulites. For this copolymer, the growth rate decreased in relative terms (see Figure 6c) by a factor of approximately 2.5 at a supercooling of 40 °C.

The data presented in Figure 6a are plotted as a function of supercooling in the Supplementary Information (Figure SI-5). The PBS-rich growth rate data is shifted horizontally and but there is no overlap in the y axis values. If we were dealing with a simple solvent effect, the growth rate curves at different compositions should completely overlap in a master curve when plotted as a function of supercooling. The lack of superposition is due to the fact that PCL repeat units are randomly incorporated and covalently bonded with the PBS repeat units. The interruption of crystallizable PBS repeat units (by the majority of PCL repeat units that are excluded from the crystals) makes more difficult the secondary nucleation process. 

Figure 6c shows how the growth rate depends on composition at a constant supercooling of 40 °C. The trend is the opposite as that obtained for primary nucleation (compare Figure 6c with Figure 4a). In order to quantify the restrictions imposed by the comonomer on the crystallization of the major component, we employed the Lauritzen and Hoffman theory, as it allows the calculation of energetic terms related to the secondary nucleation process (i.e., growth process).

The Lauritzen and Hoffman (LH) nucleation and growth theory [32] was used to fit the spherulitic growth rate data as a function of isothermal crystallization temperature, according to the following equation:

(3) $$G=G_{0}\exp\left[\frac{-U^{*}}{R\left(T_{c}-T_{0}\right)}\right]\left[\frac{-K_{g}^{G}}{fT\left(T_{m}^{0}-T_{c}\right)}\right],$$

where *G*_0_ is the growth rate constant that includes all the terms that are temperature-insensitive, U* is the transport activation energy which characterizes molecular diffusion across the interfacial boundary between melt and crystals (in this work, we employ a constant value of 1500 cal/mol). *T_c_* is the crystallization temperature and *T*_0_ is a hypothetical temperature at which all chain movements freeze (taken as *T*_0_ = *T_g_* − 30 °C); $T_{m}^{0}$ is the equilibrium melting temperature and *f* is a temperature correction factor given by the following expression: *f* = 2*T_c_*/(*T_c_* + $T_{m}^{0}$).

The equilibrium melting temperatures $T_{m}^{0}$ were estimated by the Hoffman–Weeks linear extrapolation (Figure SI-3 and Table SI-1). The parameter $K_{g}^{G}$ is proportional to the energy barrier for secondary nucleation or spherulitic growth and is given by:

(4) $$K_{g}^{G}=\;\frac{jb_{0}\sigma \sigma_{e}T_{m}^{0}}{k\Delta h_{f}},$$

where *j* is assumed to be equal to 2 for crystallization in the so-called Regime II, a regime where both secondary nucleation at the growth front and the rate of spread along the growing crystal face are comparable [26]. The other terms in the equation are the width of the chain *b_o_*, the lateral surface free energy *σ*, the fold surface free energy *σ_e_*, the Boltzman constant k, and the equilibrium latent heat of fusion, $\Delta H_{m}^{0}$. 

Plotting $lnG+\frac{-U}{R\left(Tc-T0\right)}$ versus 1/*T_c_*(Δ*T*)*f* (i.e., the Lauritzen and Hoffman plots) gives a straight line and its slope and intercept are equal to $K_{g}^{G}$ and *G*_0_ respectively. Examples of LH plots can be found in the Supplementary Information, Figure SI-6. Having the value of $K_{g}^{G}$, the magnitude of *σσ_e_* can be calculated from Equation (5). In order to calculate separately the values of *σ* and *σ_e_*, the following expression can be used [33]:

(5) $$\sigma =0.1\Delta h_{f}\sqrt{a_{0}b_{0}},$$

where *a*_0_*b*_0_ is the cross sectional area of the chain. To obtain the parameters of the LH theory, the following values were used for neat PBS and BS-rich compositions [34,35]: *a*_0_ = 5.25 Å and *b*_0_ = 4.04 Å, and for neat PCL and CL-rich compositions [36]: *a*_0_ = 4.52 Å and *b*_0_ = 4.12 Å.

Finally, *q*, the work done by the macromolecule to form a fold is given by [33]:

*q* = 2*a*_0_*b*_0_*σ_e_*. (6)

The solid lines in Figure 6a,b correspond to fittings to Equation (3). Table 2 shows that $K_{g}^{G}$ values (which are proportional to the energy barrier for spherulitic growth) for the PBS-rich crystal phase tend to increase as PCL repeat units are incorporated in the random copolymers until a maximum value is reached at the pseudo-eutectic point. Similar trends are observed for the fold surface free energy and for the work done to form folds. 

A plot of fold surface free energy versus composition can be found in the Supplementary Information (Figure SI-7). These results quantitatively measure how comonomer incorporation makes difficult the spherulitic growth of the PBS-rich phase. A similar interpretation can be done to the mirror values presented in Table 2 for PCL and the BS_11_CL_89_ copolymer with respect to the PCL phase. 

The results presented in the two sections above can be summarized by comparing Figure 4 with Figure 6. The incorporation of comonomers at each side of the eutectic causes an increase in the nucleation density and nucleation rate but at the same time a decrease in spherulitic growth rate. These two processes, primary nucleation and growth are combined when a semicrystalline polymer is crystallized from the melt. Their simultaneous effect can be ascertained by determining overall crystallization kinetics by DSC.

### 3.3. Overall Crystallization Kinetics Studied by DSC

**Figure 7:**
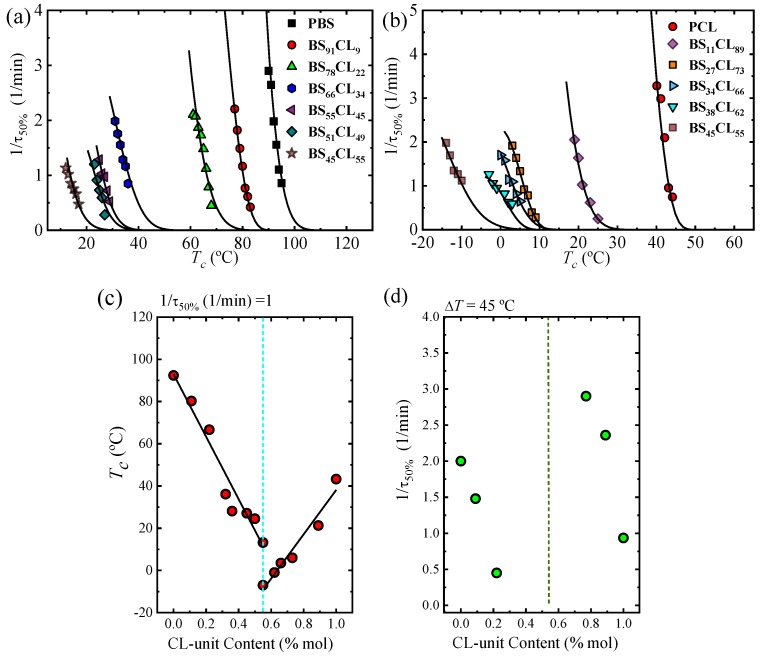
Overall crystallization versus isothermal crystallization temperature for neat PBS and PBS-rich compositions (**a**) and for neat PCL and PCL-rich compositions (**b**) versus *T_c_*. Continuous lines correspond to the fitting of the Lauritzen–Hoffman theory with the parameters in Table 3. Changes of *T_c_* versus CL-unit content in a constant rate (1/*τ*_50%_ = 1 min^−1^) (**c**). Changes of inverse of half-crystallization time, 1/*τ*_50%_ versus CL-unit content in a constant supercooling degree, Δ*T* = 45 °C (**d**).

The overall isothermal crystallization kinetics considers both nucleation and growth, and can be conveniently determined by isothermal DSC experiments. Figure 7a,b shows the experimental overall crystallization rate expressed as the inverse of the crystallization half time (*τ*_50%_). By DSC, we were able to determine the overall isothermal crystallization kinetics for both homopolymers and all copolymers (five copolymers where only the PBS-rich phase crystallized and four copolymers where only the PCL-rich phase can crystallize). 

For the special composition at the eutectic point (i.e., BS_45_CL_55_) that shows two crystalline phases, PBS-rich phase and PCL-rich phase, we performed isothermal crystallization using different protocols. For the PBS-rich phase crystallization, isothermal DSC experiments were performed at temperatures where the PCL-rich phase is in the melt and cannot crystallize, while in the PCL-rich phase, a special protocol was adopted to previously crystallize the PBS-rich phase to saturation (see experimental part).

Figure 7a,b shows the strong dependence of the overall crystallization rate and the temperature range where measurements were possible on copolymer composition. In the case of the PBS and all PBS-rich compositions, the overall crystallization proceeds from a single-phase melt. Upon increasing PCL content, the amount of the crystallizable PBS-rich phase decreases and there will be more molten PCL component causing a plasticization (“solvent effect”). In addition, the effect of PCL exclusion in the PBS-rich crystal lattice may cause some further reduction in crystallization rate. Figure 7a shows that the temperature needed for crystallization decreases as PCL content in the copolymer increases, while the overall crystallization rate measured at the minimum *T_c_* value possible tends to decrease.

Figure 7c plots the crystallization temperature needed to obtain the same overall crystallization rate of 1 min^−1^. These T_c_ values monotonically decrease with PCL content until the pseudo-eutectic region is reached. On the PCL-rich side, Figure 7b,c shows similar results, as the crystallization temperatures needed to crystallize the PCL phase decrease as PBS repeat units are added to the copolymer.

**Figure 8:**
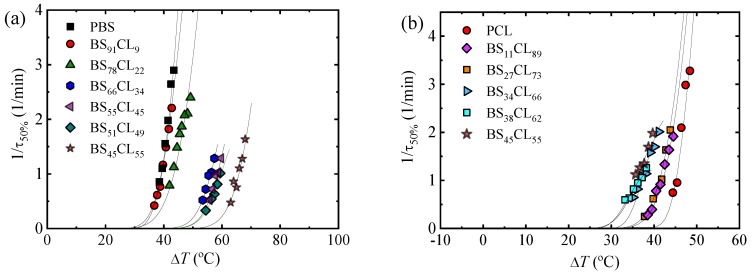
Overall crystallization for neat PBS and PBS-rich compositions (**a**) and for neat PCL and PCL-rich compositions (**b**) versus supercooling temperature.

To check if the supercooling is playing a major role upon changing composition, we plot the data contained in Figure 7a,b as a function of Δ*T* in Figure 8a,b, respectively. Surprisingly, the trends are quite different depending on the phase under consideration, or the composition range.

Figure 8a shows that the curves of PBS-rich overall growth rate data that originally spanned a *T_c_* range of approximately 90 °C (in Figure 7a) are now within 30 °C in supercooling, attesting for the thermodynamic compensation of the solvent effect, as the PCL-rich phase is in the melt. In fact, the curves of PBS and BS_91_CL_9_ completely overlap, while that of BS_78_CL_22_ is relatively close to that of neat PBS. However, beyond 22% CL incorporation in the copolymer, the samples require much larger supercooling to crystallize. It is clear that the dominant factor to the left of the eutectic point is the growth rate, as the results presented in Figure 6a and Figure 7a imply an overall crystallization rate reduction with PCL incorporation in the copolymer, both in terms of crystallization temperature or supercooling. In spite of the increase in nucleation density and nucleation rate (see Figure 3 and Figure 4) with PCL incorporation in the copolymer, it is the very large decrease in growth rate (of up to three orders of magnitude, see Figure 6b) that dominates, leading to a decrease in overall crystallization rate (Figure 7a and Figure 8a). 

Figure 7d represents the overall crystallization rate as a function of composition for a constant supercooling of 45 °C. In the PBS-rich side of the pseudo-eutectic region (left-hand side of Figure 7d), only three data points are plotted, as they are the only ones that could be measured at such constant supercooling value (check Figure 8b). The trend clearly shows a significant decrease in overall crystallization rate as CL unit content increases, as expected from Figure 7a and Figure 8a and the discussion above. Even though the value of supercooling is not exactly the same (with only 5 °C difference), a comparison with Figure 4a and Figure 6c clearly indicates that the PBS-rich phase overall crystallization is dominated by growth rate.

Figure 8b shows remarkable results for the PCL-rich phase overall crystallization. The 1/*τ*_50%_ curves versus temperature span a temperature range of 65 °C (Figure 6b). When they are plot as a function of supercooling, they only span 15 °C. However, they do not overlap, as would be expected for a simple solvent effect. In fact, the supercooling needed for crystallization of the PCL-rich phase remarkably decreases as PBS repeat units are included in the copolymer. The results indicate an acceleration of the overall crystallization rate (at constant supercooling) that can only be explained by the increase in both nucleation density and nucleation rate. We were only able to measure the increase in nucleation density and nucleation rate in Figure 3 and Figure 4 for neat PCL and BS_11_CL_89_, as further incorporation of BS units increased the nucleation density so much that measurements by polarized optical microscopy of nucleation rate became impossible. Hence, we are convinced that primary nucleation enhancement upon PBS repeat unit incorporation in the copolymers is the reason behind the acceleration of the overall crystallization kinetics, when this is considered in terms of supercooling. In the right-hand side of the pseudo-eutectic point in Figure 7d, the increase in overall crystallization rate at a constant supercooling of 45 °C can be appreciated. 

The Lauritzen and Hoffman theory can also be applied to fit the overall crystallization data presented above. Equation (3) has to be modified to employ, as a characteristic rate, the inverse of the half-crystallization time determined by DSC, as follows [37]:

(7) 1τ50%=1τ50%exp[UR(Tc−T0)][−KgτfT(Tm0−Tc)],

where all the terms have been defined above, except for $K_{g}^{\tau }$, which now represents a parameter proportional to the energy barrier for both primary nucleation and spherulitic growth. The superscript τ is used to indicate its origin (coming from DSC data, and hence from fitting 1/τ_50%_ versus crystallization temperature). In this way, it is different from $K_{g}^{G}$, defined in Equation (3), derived from growth rate data and therefore proportional just to the free energy barrier for secondary nucleation or growth. The solid lines in Figure 7a,b and Figure 8a,b represent the fits to the Lauritzen and Hoffman theory. Table 3 on the other hand reports all the relevant parameters. 

There are no results in the literature regarding the isothermal crystallization of PBS-ran-PCL copolymers, therefore, we cannot compare the parameters reported in Table 2 and Table 3 with literature values. In the case of the homopolymers, Wu et al. [38] and Papageorgiou et al. [39] reported $K_{g}^{\tau }$ values for neat PBS equal to 1.157 × 10^5^ and 2.64 × 10^5^ K^2^, respectively, which are close to the value obtained in this work, i.e., 2.04 × 10^5^ K^2^. A value for $K_{g}^{G}$ for neat PBS reported [39] equal to 1.88 × 10^5^ has been reported, that is somewhat higher than that obtained in this work, i.e., 0.87 × 10^5^ K^2^. For neat PCL, the energetic parameters previously reported based on fits of the Lauritzen and Hoffman theories [40] are in the same order of magnitude as those reported here [33].

**Figure 9:**
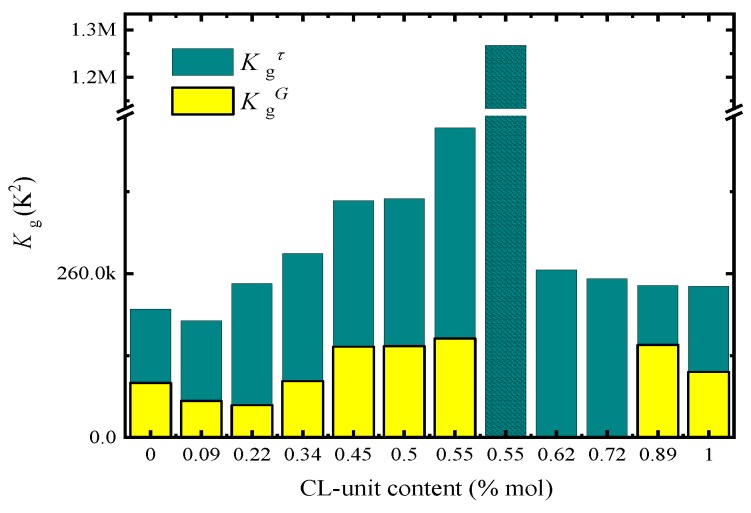
K_g_ versus CL-unit molar fraction that obtained for PLOM experiments ($K_{g\;}^{G}$) and DSC experiments ($K_{g\;}^{\tau }$).

Figure 9 plots both K_g_ values, obtained by PLOM ($K_{g\;}^{G}$) and DSC ($K_{g\;}^{\tau }$) as a function of CL-unit molar content. As expected, all $K_{g\;}^{\tau }$ values are larger than $K_{g}^{G}$ values, as DSC measurements take into account both nucleation and growth, while PLOM measurements considered only growth (see more details in [37]).

In the case of $K_{g\;}^{G}$ values, the trends observed are expected in view of the results obtained in Figure 6. The energy barrier for crystal growth increased with CL-unit molar content, since growth rate decreased as comonomer incorporation increased. On the other hand, when we analyzed the results obtained for $K_{g\;}^{\tau }$ in Figure 9, we noticed that there is a clear asymmetry depending on which side of the pseudo-eutectic region the material is. On the PBS-rich side (left-hand side of Figure 9), $K_{g\;}^{\tau }$ values rapidly increased upon CL units addition. This is expected from the results presented in Figure 7d, where a large decrease in overall crystallization rate for the PBS-rich side of the composition range can be observed. 

In the case of the PCL-rich composition range, we would have expected a decrease in $K_{g\;}^{\tau }$ values with PCL content increases according to Figure 7d. Instead, we observe in Figure 9 that the energy barrier for both nucleation and growth does not significantly change with composition (see right-hand side of Figure 9). We have to remember that for the PCL-rich copolymers the situation is particularly complicated as the nucleation density and nucleation rate increase with CL-unit content but the growth rate decreases. Hence, even though according to Figure 7d the overall crystallization rate at constant supercooling seems to be dominated by primary nucleation, the values of $K_{g\;}^{\tau }$ are obtained from the slope of the Lauritzen and Hoffman plots that take into account the full range of supercoolings where the measurements were taken. Therefore, it seems that when the overall energy barrier is considered, there is a balance between nucleation and growth which keeps the $K_{g\;}^{\tau }$ values constant with composition.

### 3.4. Double Crystallization at the Pseudo-Eutectic Point

**Figure 10:**
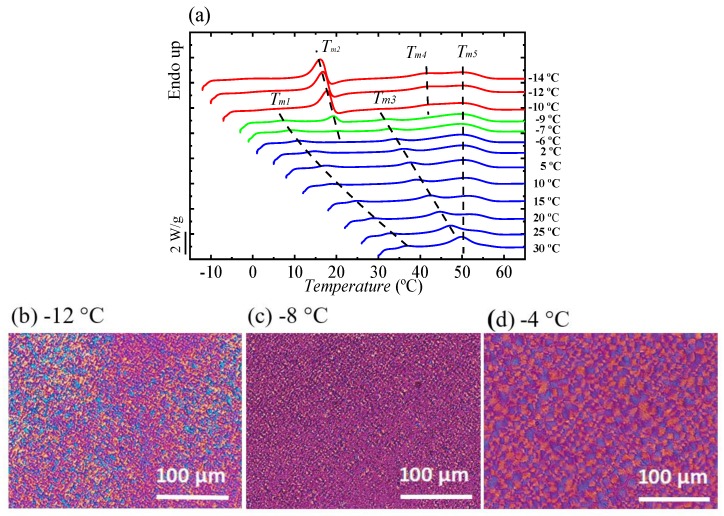
(**a**) DSC heating runs (at 10 °C/min) after isothermal crystallization at different temperatures. See text for the explanation of the color code employed. PLOM micrographs after isothermal crystallization at −12 °C (**b**), at −8 °C (**c**), and at −4 ° C (**d**) for the BS_45_CL_55_ sample.

For the copolymer whose composition is within the pseudo-eutectic point, i.e., BS_45_CL_55_, we performed isothermal crystallization in a wide range of crystallization temperatures *T_c_*, to find the temperature region where only one of the phases, PBS or PCL, is able to crystallize. Figure 10 shows the heating DSC scan recorded at 10 °C/min for BS_45_CL_55_ sample after it was isothermally crystallized at the indicated *T_c_* values.

At least five different endotherms can be found upon close examination of Figure 10 and we indicated with dashed lines how these endotherms approximately shift depending on the *T_c_* values employed before heating the samples. The first melting peak *T*_m1_ is present in all melting curves and its location is almost at 7 °C higher than the crystallization temperature. This peak has been traditionally regarded as the melting of thin crystals formed during the secondary crystallization process [41]. The second peak, labeled *T*_m2_, appeared at *T_c_* values lower than −6 °C and corresponds to the melting of PCL-rich crystals. The third peak or *T*_m3_ labeled peak in Figure 10 highly depends on the isothermal crystallization temperature and corresponds to the melting of the PBS-rich crystals, which were formed during the isothermal crystallization. 

In addition, a melting peak (*T*_m5_) at around 50 °C and another one just below it (*T*_m4_) were observed. The melting peak labeled *T*_m5_ corresponds to the melting of PBS crystals that have reorganized during the heating scan, and have a melting point which is almost constant at around 50 °C, regardless of the crystallization temperature [42,43]. The *T*_m2_ and *T*_m3_ peaks increase almost linearly with increasing *T_c_*. As shown in Figure 10, *T*_m3_ disappeared in the DSC heating curves where the crystallization temperature is less than −9 °C. 

The morphologies obtained after isothermal crystallization at three selected temperatures can be observed in Figure 10b–d. As it will be shown below, WAXS experiments confirmed that at very low *T_c_* values including −12 °C, only PCL-rich crystals can be formed. Figure 10d shows small spherulites that were formed at *T_c_* = −4 °C with spherulites size around 10 µm. At *T_c_* = −8 °C, where both PCL and PBS crystals can form, there are two crystals sizes, one with 4 µm radii (PBS crystals) and another with around 1.5 µm size (PCL crystals), see Figure 10d. Figure 10b shows only PCL crystals with small spherulites size (less than 1 µm) at *T_c_* = −12 °C.

We performed in situ synchrotron WAXS experiments for the sample at the pseudo-eutectic point, to clarify the temperature range of crystallization of the PBS-rich and the PCL-rich phases and corroborate the assignment of the thermal transitions in Figure 10. These experiments were performed during isothermal crystallization (for 20 min) at three different *T_c_* values chosen from three different crystallization regions in Figure 10. 

**Figure 11:**
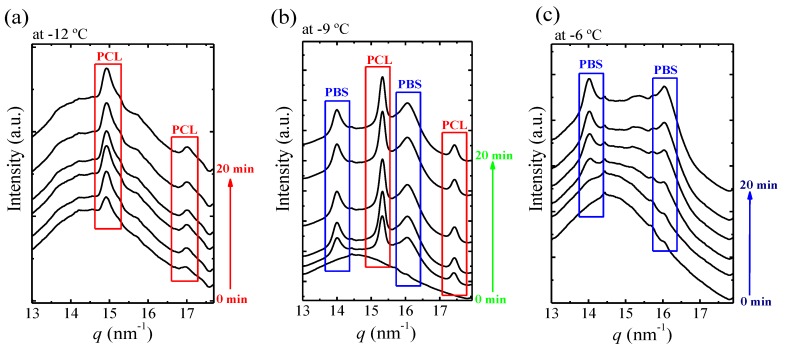
Wide angle X-ray diffraction (WAXS) diffraction patterns of BS_45_CL_55_ registered during isothermal crystallization at −12 °C (**a**), −9 °C (**b**), and −6 °C (**c**).

Figure 11a–c shows selected real-time WAXS diffractograms for BS_45_CL_55_ (i.e., the sample at the pseudo-eutectic point) measured during isothermal crystallization at −12, −9, and −6 °C. If the sample shows characteristic reflections at q = 13.9 and 16.1 nm^−1^, they correspond to the PBS (020) and (110) crystallographic planes. If the sample exhibits reflections at q = 15.3 and 17.4 nm^−1^, they belong to the PCL (110) and (200) planes [10].

Changes in the crystallization temperature strongly affect the diffraction pattern at the pseudo-eutectic point. As can be seen in Figure 11, at −12 °C only the PCL-rich phase is able to crystalize (Figure 11a) while at −6 °C (Figure 11c) only the PBS-rich phase crystallizes. On the other hand, at the intermediate *T_c_* value of −9 °C, both PBS-rich and PCL-rich phases can crystallize. If the DSC curves of Figure 10 are considered again, the WAXS assignments are consistent with the heating runs after crystallization for all samples crystallized at −9 °C and higher. In the case of low crystallization temperatures, i.e., below −9 °C, it should be noted that WAXS indicate that only the PCL-rich phase can crystallize. The DSC heating runs shown in Figure 10 also show melting transitions corresponding to the melting of PBS-rich phase. These PBS-rich phase crystals must be formed by cold-crystallization during the heating scan for the samples crystallized at −10, −12, and −14 °C in Figure 10. In fact, upon close examination of Figure 10, the end of a cold crystallization process can be observed just after the melting peak of the PCL-rich phase crystals.

Taking into account the WAXS and DSC results presented in Figure 10 and Figure 11, the DSC curves in Figure 10 were plotted with a color code to indicate which phases can crystallize during isothermal crystallization depending on the *T_c_* values employed. If the *T_c_* values are −10 °C or lower, only the PCL-rich phase can crystallize, and the curves were arbitrarily plotted in red in Figure 10a. If the *T_c_* values are between −9 and −7 °C (including these two temperatures), both the PCL-rich and the PBS-rich phases can crystallize (green curves in Figure 10). Finally, if the *T_c_* temperatures are −6 °C and above, only the PBS-rich phase can crystallize (blue curves in Figure 10). 

The pseudo-eutectic sample, BS_45_CL_55_, exhibits a very interesting phase behavior, as depending on the crystallization conditions, one or both phases can be formed. We have studied previously the nonisothermal crystallization of the same copolymers employed here [17]. It is interesting to note that under nonisothermal conditions, the cooling rate employed determines which phase can crystallize and also if one or two phases are formed. In this work, on the other hand, we show that one or two phases can be formed depending on the isothermal crystallization temperature chosen. Therefore, the properties of this isodimorphic copolyester with pseudo-eutectic composition can be tailored by varying both nonisothermal or isothermal crystallization conditions, a remarkable and novel behavior, as far as the authors are aware.

## 4. Conclusions

The complex isothermal nucleation, growth and overall crystallization of isodimorphic PBS-*ran*-PCL copolyesters were studied for the first time. The equilibrium melting temperatures show a very clear pseudo-eutectic point at a composition of BS_45_CL_55_. To the left of the pseudo-eutectic point (in a plot of *T_m_^0^* versus CL-unit molar content) only the PBS-rich phase is able to crystallize, at the pseudo-eutectic point both PBS-rich and PCL-rich phases can crystallize and to the right of the pseudo-eutectic point only PCL-rich crystals are formed. With respect to the parent homopolymers, any comonomer incorporation on either side of the pseudo-eutectic point causes as increase in nucleation density and nucleation rate, as well as a decrease in spherulitic growth rate. 

As a result, the overall crystallization rate determined by DSC was a strong function of composition and supercooling. For PBS-rich copolymers, the PBS-rich phase overall crystallization rate-determining-step was the spherulitic growth rate. On the other hand, for PCL-rich copolymers, the nucleation rate (which was always larger for mirror compositions) gained more importance in the control of the overall crystallization rate. 

The crystallization of the isodimorphic copolyester with pseudo-eutectic composition can be tailored by varying the isothermal crystallization temperature, depending on which, either one or both phases are able to crystallize. Such remarkable property control allows the possibility of having a single copolyester with only PCL crystals, only PBS crystals, or both types of crystals, thus exhibiting very different thermal properties. 

## Figures and Tables

**Table 1:** Molar composition determined by ^1^H-NMR, number- and weight-average molar mass determined by gel permeation chromatography (GPC), and thermal transitions determined by differential scanning calorimetry (DSC) (at 10 °C/min) of the materials employed in this work.

	Copolyester	*M_n_*	*M_w_*	*T_g_* (°C)	*T_c_* (°C)	*T_m_* (°C)
1	PBS	7500	21,470	−33	76	114
2	BS_91_CL_9_	8790	21,640	−36	61	104
3	BS_78_CL_22_	6580	18,000	−40	46	93
4	BS_66_CL_34_	7830	19,700	−44	14	73
5	BS_62_CL_38_	9750	27,300	−45	12	70
6	BS_55_CL_45_	8970	24,700	−46	7	62
7	BS_51_CL_49_	7400	23,500	−47	4	58
8	BS_45_CL_55_	8000	17,300	−48	−5/−22	13/49
9	BS_38_CL_62_	11,000	24,300	−50	−13	17
10	BS_34_CL_66_	10,000	29,900	−51	−10	18
11	BS_27_CL_73_	11,540	28,700	−53	−8	19
12	BS_11_CL_89_	6300	19,500	−56	10	36
13	PCL	5400	17,400	−60	34	55

**Table 2:** Primary nucleation and growth isothermal kinetics data parameters according to Equations (1) and (3) derived from experimental results obtained by PLOM.

Copolyester	Nucleation, Equation (1)		Growth, Equation (3)				
	Δ*σ* (erg/cm^2^)	*^a^R* ^2^	$K_{g}^{G}\;(K^{2})$	*σ* (erg/cm^2^)	*σ_e_* (erg/cm^2^)	*q* (erg)	*^b^R* ^2^
PBS	1.97	0.938	8.66 × 10^4^	12.4	79.5	3.37 × 10^−13^	0.983
$BS_{91}CL_{9}^{\;}$	1.55	0.963	5.80 × 10^4^	12.4	55.0	2.33 × 10^−13^	0.994
$BS_{78}CL_{22}^{\;}$	1.52	0.993	5.12 × 10^4^	12.4	49.6	2.10 × 10^−13^	0.973
$BS_{66}CL_{34}^{\;}$	0.58	0.977	8.94 × 10^4^	12.4	88.9	3.78 × 10^−13^	0.982
$BS_{62}CL_{38}^{\;}$	0.36	0.913	8.92 × 10^4^	12.4	89.4	3.87 × 10^−13^	0.982
$BS_{55}CL_{45}^{\;}$	0.44	0.920	14.4 × 10^4^	12.4	148.8	6.30 × 10^−13^	0.999
$BS_{51}CL_{49}^{\;}$	0.32	0.944	14.5 × 10^4^	12.4	148.9	6.32 × 10^−13^	0.999
$BS_{45}CL_{55}^{\;}$	0.14	0.973	15.7 × 10^4^	12.4	165.5	7.02 × 10^−13^	0.974
$BS_{11}CL_{89}^{\;}$	1.13	0.867	14.7 × 10^4^	6.8	169.6	6.32 × 10^−13^	0.999
PCL	1.53	0.999	10.4 × 10^4^	6.8	112.0	4.17 × 10^−13^	0.996

*^a^ R*^2^ is the correlation coefficient for the fitting of the nucleation kinetics with the Turnbull–Fisher model (Equation (1)), *log I* vs. 1/*T*(Δ*T*)^2^. *^b^ R*^2^ is the correlation coefficient for the fitting of the Lauritzen–Hoffman model (Equation (3)), *lnG* + U*/*R*(*T_c_* − *T*_0_) vs. 1/*f.Tc.*Δ*T*.

**Table 3:** Parameters obtained from fitting the DSC data presented in Figure 7a,b to the Lauritzen and Hoffman model (Equation (7)).

Copolyester	$K_{g}^{\tau }\;(K^{2})$	*R* ^2^	*σ* (erg/cm^2^)	*σ_e_* (erg/cm^2^)	*q* (erg)
PBS	2.04 × 10^5^	0.9697	12.4	186.9	7.93 × 10^−13^
$BS_{91}CL_{9}^{\;}$	1.85 × 10^5^	0.9938	12.4	173.8	3.37 × 10^−13^
$BS_{78}CL_{22}^{\;}$	2.44 × 10^5^	0.9514	12.4	236.3	10.0 × 10^−13^
$BS_{66}CL_{34}^{\;}$	2.92 × 10^5^	0.9958	12.4	290.4	12.3 × 10^−13^
$BS_{55}CL_{45}^{\;}$	3.76 × 10^5^	0.9775	12.4	387.3	16.4 × 10^−13^
$BS_{51}CL_{49}^{\;}$	3.79 × 10^5^	0.9973	12.4	388.5	16.8 × 10^−13^
$BS_{45}CL_{55}^{\;}\left(BS-rich\right)$	4.91 × 10^5^	0.9502	12.4	518.7	22.0 × 10^−13^
$BS_{45}CL_{55}^{\;}\left(CL-rich\right)$	12.5 × 10^5^	0.9880	6.8	1377.0	51.3 × 10^−13^
$BS_{38}CL_{62}^{\;}$	2.66 × 10^5^	0.9917	6.8	328.0	12.6 × 10^−13^
$BS_{27}CL_{73}^{\;}$	2.52 × 10^5^	0.9845	6.8	305.9	11.4 × 10^−13^
$BS_{11}CL_{89}^{\;}$	2.41 × 10^5^	0.9945	6.8	278.4	10.4 × 10^−13^
PCL	2.40 × 10^5^	0.9082	6.8	260.6	9.71 × 10^−13^

*R*^2^ is the correlation coefficient for the Lauritzen–Hoffman (Equation (7)) linear plots ln(1/*τ* 50%) + U*/*R*(*T_c_* − *T*_0_) vs. 1/*f*·*T_c_*·Δ*T*.

## Supplementary Materials

The following materials are available online at https://www.mdpi.com/2073-4360/12/1/17/s1, Table SI-1. Equilibrium melting temperatures for CoP(BS_x_CL_y_) compositions and their corresponding homopolymers, Figure SI-1. Nucleation kinetics studies by PLOM. Nuclei density as a function of time at different crystallization temperature for PBS-rich phase samples: (a) BS_91_CL_9_, (b) BS_66_CL_34_, (c) BS_62_CL_38_, (d) BS_55_CL_45_, (e) BS_51_CL_49_, and (f) BS_45_CL_55_. *T_c_* employed are chosen so that Δ*T* = 40, 38, 36, 34, 32 °C for all samples, Figure SI-2. Nuclei density during isothermal crystallization as a functional Δ*T* for PBS-rich (a) and for PCL-rich (b) copolyesters, Figure SI-3. Hoffman–Weeks plots for PBS-*ran*-PCL compositions. The black solid line represents the thermodynamic equilibrium line *T_m_ = T_c_*, Figure SI-4. Plot of *log I* versus 1/*T*(Δ*T*)^2^ and fitting to Turnbull–Fisher equation (Equation (1)) for PBS-rich (a) and PCL-rich (b) compositions, Figure SI-5. Spherulitic growth rates *G* determined by PLOM for neat PBS and PBS-rich (a) and for neat PCL and PCL-rich (b) copolymers as a function of supercooling, Figure SI-6. The fits to the Lauritzen–Hoffman equation using the free Origin plug-in developed by Lorenzo et al. and the experimental data for the (a-a’) PBS, (b-b´) BS_78_CL_22_, and (c-c´) BS_45_CL_55_, Figure SI-7. The *σ_e_* value versus CL-unit molar fraction that obtained for PLOM experiments ($\sigma_{g\;}^{G}$) and DSC experiments ($\sigma_{g\;}^{\tau }$).
