# Supplementary material for: Controlling the Isothermal Crystallization of Isodimorphic PBS-ran-PCL Random Copolymers by Varying Composition and Supercooling
Source: Polymers (Basel). 2019 Dec 20;12(1):17. doi: 10.3390/polym12010017 (PMC7022771; doi:10.3390/polym12010017)
Supplement: Supplementary file 1 [file polymers-12-00017-s001.pdf]

Supporting Information

Controlling the Isothermal Crystallization of Isodimorphic PBS-*ran*-PCL Random Copolymers by Varying Composition and Supercooling

by Maryam Safari[1], Agurtzane Mugica[1], Manuela Zubitur[2], Antxon Martínez de Ilarduya[3], Sebastián Muñoz-Guerra[3], Alejandro J. Müller *[1,4]

[1] POLYMAT and Polymer Science and Technology Department, Faculty of Chemistry, University of the Basque Country UPV/EHU, Paseo Manuel de Lardizabal 3, Donostia-San Sebastián, 20018, Spain.

[2] Chemical and Environmental Engineering Department, Polytechnic School, University of the Basque Country UPV/EHU, Plaza Europa 1, Donostia-San Sebastián, 20018, Spain.

[3] Departament d'Enginyeria Química, Universitat Politècnica de Catalunya, ETSEIB, Diagonal 647, Barcelona, 08028, Spain.

[4] IKERBASQUE, Basque Foundation for Science, María Díaz Haroko Kalea, 3, Bilbao, 48013, Spain.

* Correspondence: alejandrojesus.muller@ehu.es; Tel.: +34 943018191

Table SI-1. Equilibrium melting temperatures for CoP(BS_xCL_y) compositions and their corresponding homopolymers.

Copolyester	T_m^0 (°C)
**PBS**	131
**$BS_{91}CL_9$**	118
**$BS_{78}CL_{22}$**	110
**$BS_{66}CL_{34}$**	100
**$BS_{62}CL_{38}$**	98
**$BS_{55}CL_{45}$**	87
**$BS_{51}CL_{49}$**	85
**$BS_{45}CL_{55}$(BS-rich)**	78
**$BS_{45}CL_{55}$(CL-rich)**	35
**$BS_{38}CL_{62}$**	38
**$BS_{34}CL_{66}$**	42
**$BS_{27}CL_{73}$**	47
**$BS_{11}CL_{89}$**	63
**PCL**	88

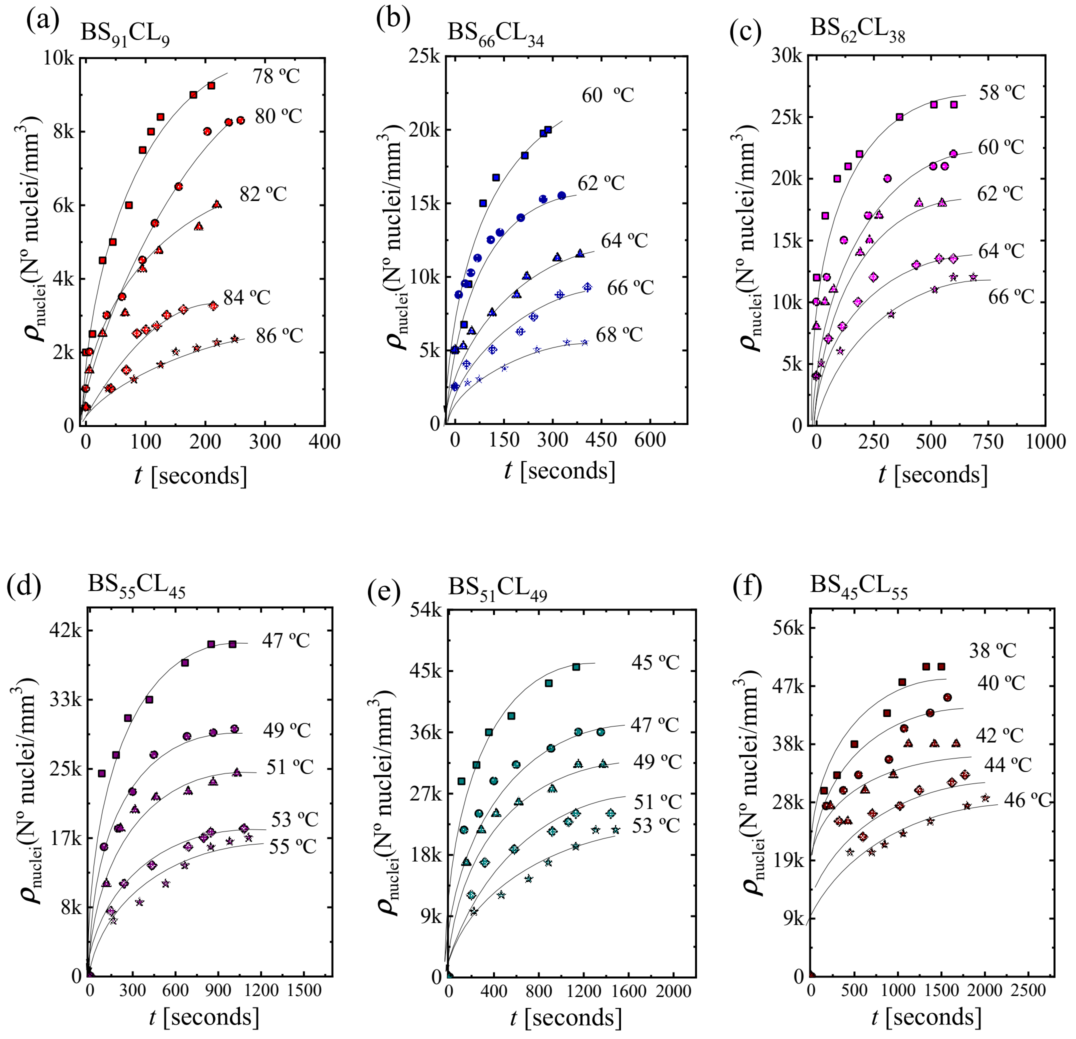

Figure SI-1. Nucleation kinetics studies by PLOM. Nuclei density as a function of time at different crystallization temperature for PBS-rich phase samples: (a) $BS_{91}CL_{9}$, (b) $BS_{66}CL_{34}$, (c) $BS_{62}CL_{38}$, (d) $BS_{55}CL_{45}$, (e) $BS_{51}CL_{49}$, and (f) $BS_{45}CL_{55}$. T_c employed are chosen so that ΔT = 40, 38, 36, 34, 32 °C for all samples.

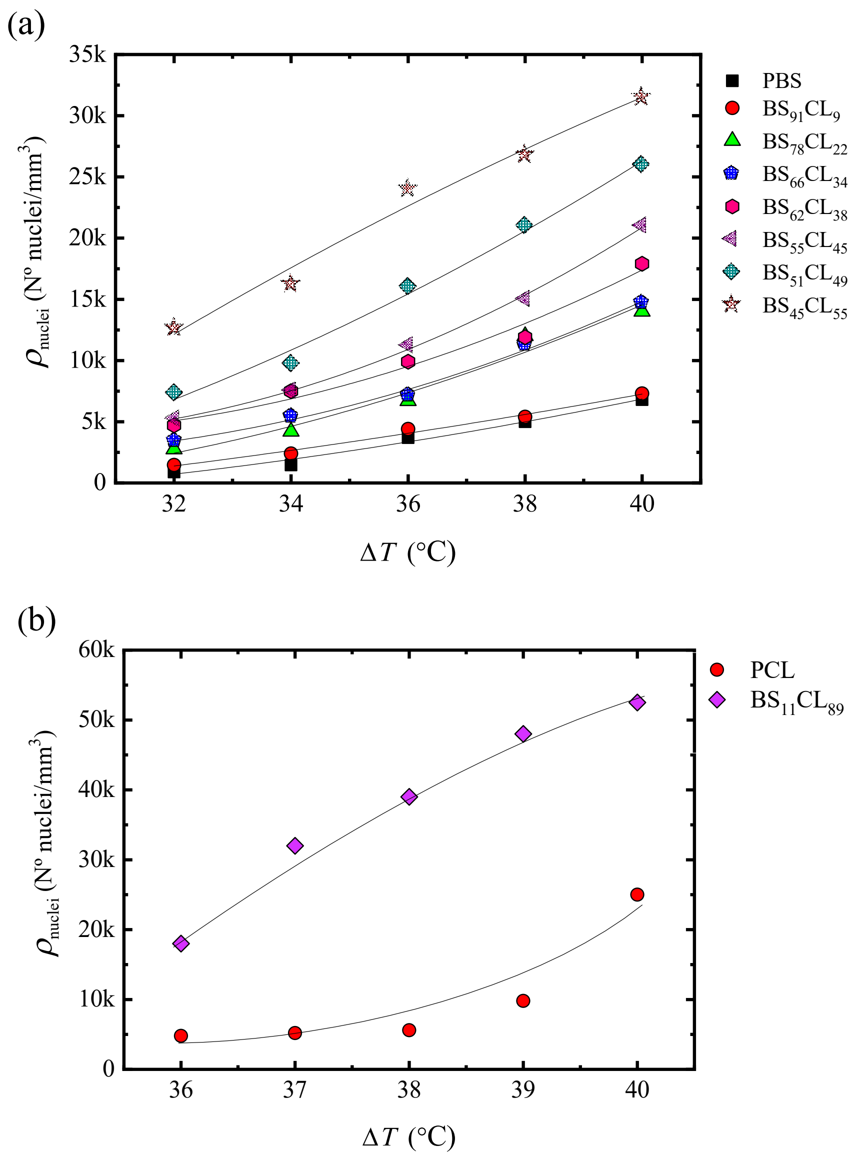

Figure SI-2. Nuclei density during isothermal crystallization as a function of ΔT for PBS-rich (a) and for PCL-rich (b) copolyesters.

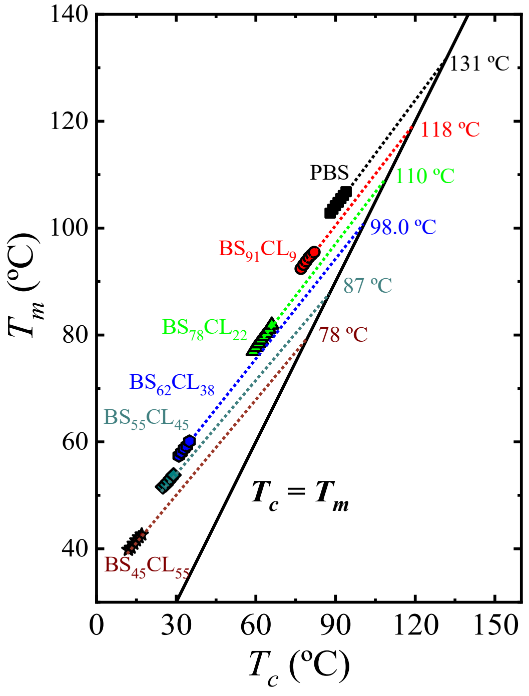

Figure SI-3. Hoffman–Weeks plots for PBS-*ran*-PCL compositions. The black solid line represents the thermodynamic equilibrium line T_m=T_c.

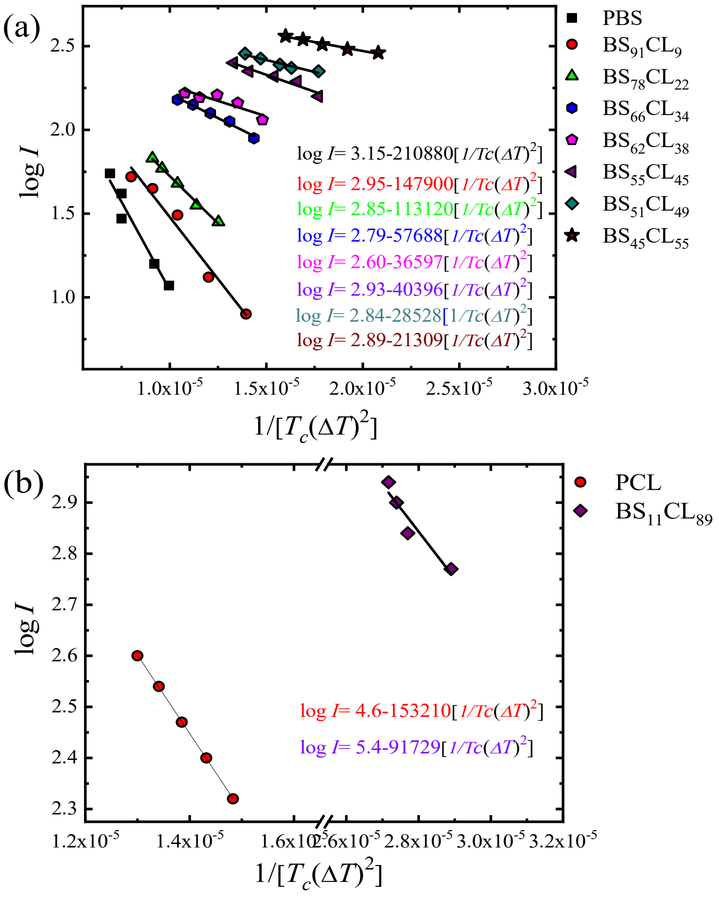

Figure SI-4. Plot of *log I* versus $1/T(\Delta T)^2$ and fitting to Turnbull–Fisher equation (Eq. 1) for PBS-rich (a) and PCL-rich (b) compositions.

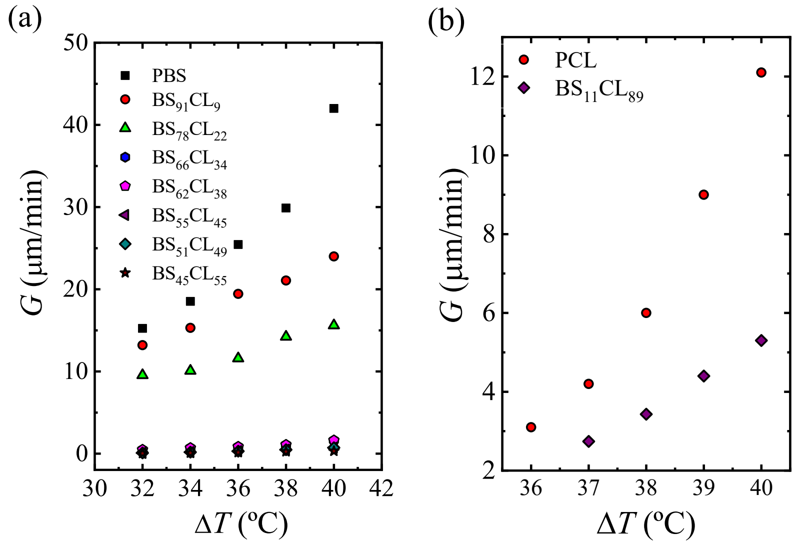

Figures SI-5. Spherulitic growth rates *G* determined by PLOM for neat PBS and PBS-rich (a) and for neat PCL and PCL-rich (b) copolymers as a function of supercooling.

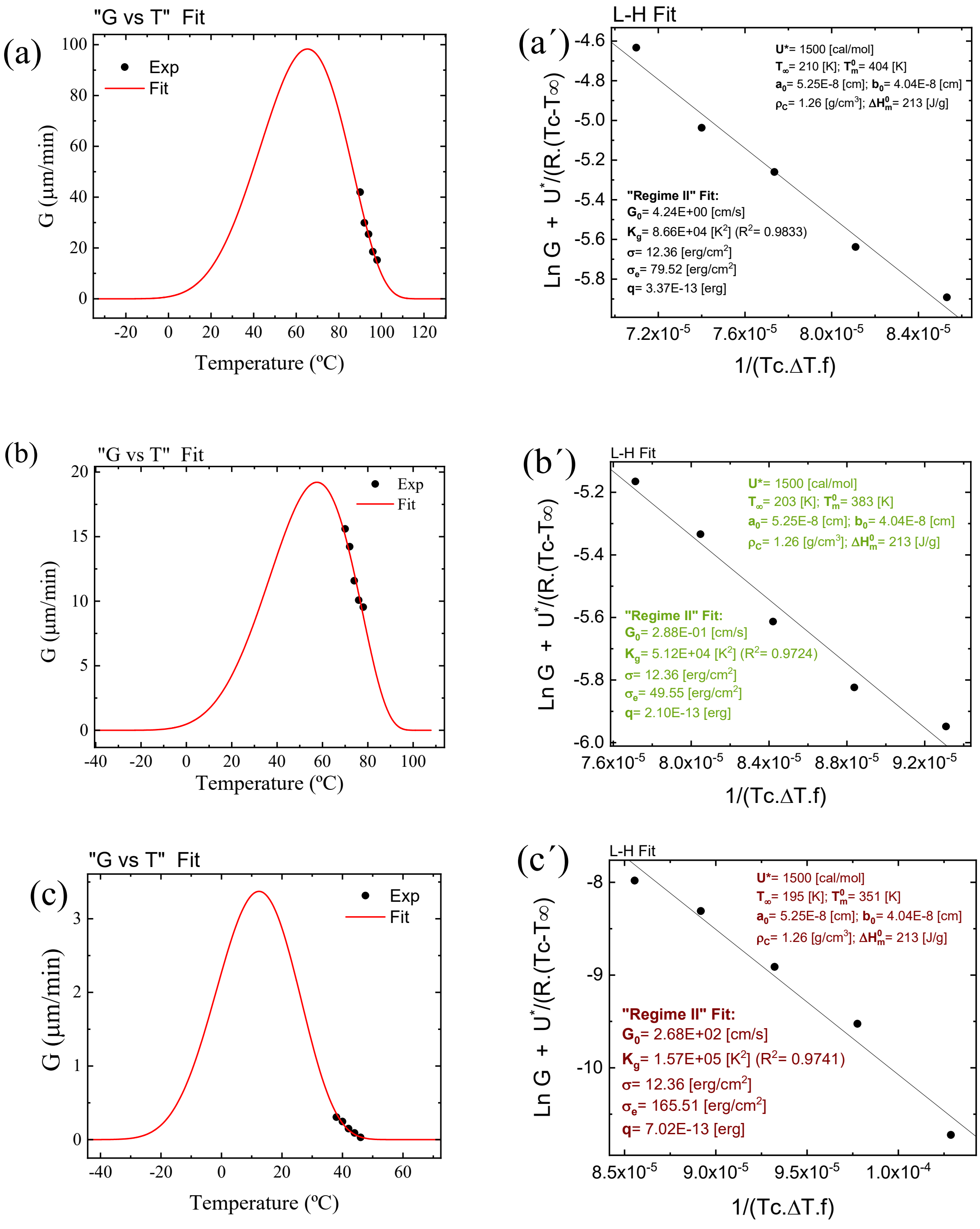

Figure SI-6. The fits to the Lauritzen-Hoffman equation using the free Origin plug-in developed by Lorenzo et al. [1] and the experimental data for the (a-a´) PBS, (b-b´) $BS_{78}CL_{22}$, and (c-c´) $BS_{45}CL_{55}$.

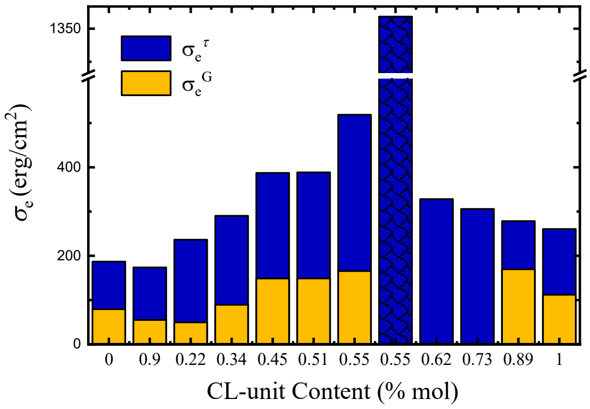

Figure SI-7. The σ_e value versus CL-unit molar fraction that obtained for PLOM experiments (σ_e^G) and DSC experiments (σ_e^τ).

References

1. Lorenzo, A. T.; Arnal, M. L.; Albuerne, J.; Müller, A. J., DSC isothermal polymer crystallization kinetics measurements and the use of the Avrami equation to fit the data: Guidelines to avoid common problems. Polymer testing 2007, 26 (2), 222-231.
